# Collateral damage of using colistin in hospitalized patients on emergence of colistin-resistant *Escherichia coli* and *Klebsiella pneumoniae* colonization and infection

**DOI:** 10.1186/s13756-018-0375-4

**Published:** 2018-07-17

**Authors:** W. Wangchinda, N. Pati, N. Maknakhon, C. Seenama, S. Tiengrim, V. Thamlikitkul

**Affiliations:** 1 Division of Infectious Diseases and Tropical Medicine, Department of Medicine, Faculty of Medicine Siriraj Hospital, Mahidol University, 2 Wanglang Road, Bangkoknoi, Bangkok, 10700 Thailand; 2 Department of Clinical Microbiology and Applied Technology, Faculty of Medical Technology, Mahidol University, Bangkok, Thailand

**Keywords:** Thailand, Prevalence, Colonization, Colistin-resistant *Escherichia coli*, Colistin-resistant *Klebsiella pneumoniae*, Colistin, Hospitalization

## Abstract

**Background:**

Colistin has been used for therapy of carbapenem-resistant Gram-negative infections in Thailand, especially carbapenem-resistant *A. baumannii* and *P. aeruginosa*, for more than 10 years. However, the prevalence of colistin-resistant *A. baumannii* or *P. aeruginosa* is still less than 5%. Colistin-resistant Enterobacteriaceae has been increasingly reported globally over the past few years and the use of colistin in food animals might be associated with an emergence of colistin resistance in Enterobacteriaceae. This study aimed to determine the effect of colistin exposure in hospitalized patients who received colistin on development of colistin-resistant (CoR) *Escherichia coli* (EC) or *Klebsiella pneumoniae* (KP) colonization and infection.

**Methods:**

A prospective observational study was performed in adult hospitalized patients at Siriraj Hospital who received colistin for treatment of infections during December 2016 and November 2017. The surveillance culture samples were collected from the stool and the site of infection of each patient who received colistin at the study enrollment, days 3 and 7 after the study enrollment, and once a week thereafter for determination of CoR EC and CoR KP. CoR EC and CoR KP were also tested for a presence of mcr-1 gene.

**Results:**

One hundred thirty-nine patients were included. Overall prevalence of CoR EC or CoR KP colonization was 47.5% among 139 subjects. Prevalence of CoR EC or CoR KP colonization was 17.3% of subjects at study enrollment, and 30.2% after study enrollment. Use of fluoroquinolones, aminoglycosides, and colistin was found to be significantly associated with CoR EC or CoR KP colonization. The mcr-1 gene was detected in 13.0% of CoR EC or CoR KP isolates, and in 27.3% of subjects with CoR EC or CoR KP colonization. CoR EC or CoR KP colonization persisted in 65.2% of the subjects at the end of the study. Five patients with CoR KP infections received combination antibiotics and they were alive at hospital discharge.

**Conclusions:**

Prevalence of CoR EC or CoR KP colonization in hospitalized patients receiving colistin was high and it was associated with the use of colistin. Therefore, patients who receive colistin are at risk of developing CoR EC or CoR KP colonization and infection.

## Background

Colistin is one of the last-resort antibiotics available for treatment of infections caused by multidrug-resistant Gram-negative bacteria, including carbapenem*-*resistant *Acinetobacter baumannii*, carbapenem*-*resistant *Pseudomonas aeruginosa*, and carbapenem*-*resistant Enterobacteriaceae (CRE) [1]. Colistin is currently in wide use as a result of the increasing prevalence of infections caused by these bacteria. Moreover, colistin is widely used in livestock for prevention, control, and treatment of infections [2]. The prior use of colistin in humans and animals is associated with colistin resistance and/or increased colistin minimal inhibitory concentration (MIC) in Gram-negative bacteria [3–5]. A main mechanism of resistance to colistin in Gram-negative bacteria is mediated by modification of lipid A, which results in a reduction in colistin affinity for Gram-negative bacteria. Colistin resistance mechanisms are usually mediated by chromosome-encoded mutations in the PmrAB and PhoPQ two-component regulatory systems, or by MgrB inactivation [6]. The emergence of a plasmid-mediated gene (mcr-1) that encodes a protein conferring resistance to colistin in Enterobacteriaceae isolated from foods, animals, and patients was reported from China in 2015 [7]. Subsequently, mcr-1-producing colistin-resistant Enterobacteriaceae have been reported from many countries around the World [8]. Colistin use in food animals is suspected as the major driver of the emergence of this plasmid-mediated colistin-resistant gene [7]. Association between colistin use for therapy of carbapenem-resistant *A. baumannii* and *P. aeruginosa* infections in humans and the emergence of colistin-resistance in these bacteria has been reported [9–13]. However, the prevalence of and association between colistin use for therapy of carbapenem-resistant Gram-negative bacterial infections in humans and the emergence of colistin resistance in Enterobacteriaceae in individuals who receive colistin are not well-understood.

Accordingly, the aim of this study was to determine the effect of colistin use in hospitalized patients on emergence of colistin-resistant (CoR) *E. coli* (EC) and *K. pneumoniae* (KP) colonization and infection.

## Methods

This prospective observational study was conducted at Siriraj Hospital, a 2300-bed university-based tertiary care hospital in Bangkok, Thailand, during December 2016 to November 2017. The study protocol was approved by the Siriraj Institutional Review Board, and written informed consent was obtained from all subjects.

Adult patients hospitalized in the medical wards or medical intensive care units at Siriraj Hospital who received colistin were included in this study. The decision to prescribe colistin treatment was made by each patient’s attending physician. Each enrolled subject had a first surveillance stool or rectal swab culture sample collected as soon as possible within several days after receiving colistin. Subsequent stool or rectal swab samples were collected on days 3 and 7 after colistin treatment, and once a week thereafter until the patient left the hospital, died, or up to 4 weeks after receiving colistin if the previously collected samples did not reveal colistin-resistant (CoR) *E. coli* (EC) or *K. pneumoniae* (KP). Surveillance culture samples from the site of infection that precipitated the use of colistin therapy were also periodically collected if they were available. Collected surveillance culture samples were grown on a MacConkey agar plate. The lactose-fermenter bacterial colonies grown on primary MacConkey agar plate after overnight incubation at 35 °C with different morphology were subcultured. Identification of the targeted bacteria (EC and KP) was made by using traditional biochemical tests including growth on MacConkey agar, Kovac’s oxidase, triple sugar iron agar reaction, indole production, methyl red, Voges-Proskauer, citrate utilization, production of hydrogen sulfide, urea hydrolysis, phenylalanine deaminase, lysine decarboxylase, ornithine decarboxylase, motility, malonate utilization, gas from glucose fermentation, lactose fermentation, manitol fermentation [14]. The minimum inhibitory concentration (MIC) of colistin was performed by broth microdilution method, and a colistin MIC ≥4 mg/L was considered colistin resistance. The susceptibility of isolated CoR EC and CoR KP to ceftriaxone and ertapenem was also assessed by disk diffusion method. Determination of presence of the mcr-1 gene was performed in CoR EC and CoR KP isolates using polymerase chain reaction (PCR) amplification [7]. Screening for the mcr-1 gene was performed by PCR with forward (5′-GTGTGGTACCGACGCTCGG-3′) and reverse (5′-CAAGCCCAATCGGCGCATC-3′) primers. PCR amplification was performed using i-Taq DNA polymerase under the following cycling conditions: 34 cycles of 94 °C for 20 s, 50 °C for 20 s, and 72 °C for 30 s, followed by 1 cycle of 72 °C for 5 min. PCR products were separated on a 1% agarose gel. The PCR amplicon size was approximately 460 bp.

Data collected from each subject included demographics, underlying diseases and conditions, type of isolated bacteria (true pathogen or colonizer), type of infection, previous antimicrobial treatment received during the 3-month period prior to study enrollment, antimicrobial treatment during the study period, and the outcome of colistin treatment. The dose, frequency and duration of colistin were also recorded.

The primary outcomes of this study were prevalence of colonization with CoR EC or CoR KP in subjects who received colistin during the study period, and the factors associated with CoR EC or CoR KP colonization. Colonization of CoR EC or CoR KP was defined as detection of CoR EC or CoR KP in surveillance culture samples in the absence of clinical features of infection at the site of the sample collection. CoR EC or CoR KP infection was defined according to the diagnosis of the patient’s attending physician. The secondary outcomes were the clinical courses of colonization with CoR EC or CoR KP in patients who received colistin during the study period, the prevalence of the mcr-1 gene in isolated CoR EC and CoR KP, and the clinical courses of subjects with CoR EC or CoR KP colonization and infection.

### Sample size calculation and data analysis

An overall colonization and infection rate of colistin-resistant Gram-negative bacteria in a cohort of critically-ill patients in intensive care units was 27% [3]. Therefore, it was estimated that at least 30% of subjects who received colistin would develop colonization with CoR EC or CoR KP during the study period. Using a variation in prevalence of developing CoR EC or CoR KP colonization during the study period of 8% and an acceptable type 1 two-sided error of 5%, a minimum sample size of 127 subjects was needed.

All data analyses were performed using SPSS Statistics version 16.0 (SPSS, Inc., Chicago, IL, USA). Demographics and clinical characteristics of patients were summarized using descriptive statistics. Categorical data are presented as frequency and percentage, and continuous data are shown as mean ± standard deviation or median and range. Comparison of categorical data was performed using chi-square test or Fisher’s exact test, with Student’s t-test used to compare continuous data. A *p*-value less than or equal to 0.05 was considered statistically significant.

## Results

### Study population

A total of 139 subjects were included. All enrolled patients received colistin, all had ≥2 stool or rectal swab samples collected, and all had their first sample collected within 5 days after the start of colistin treatment. The demographics and clinical characteristics of subjects are summarized in Table 1.

**Table 1:** Demographics and clinical characteristics of 139 subjects

Variables	Values
Female gender, n (%)	70 (50.4%)
Age (yrs), mean ± SD	67.2 ± 15.9
Comorbid conditions, n (%)	131 (94.2%)
Diabetes mellitus	53 (38.1%)
Cardiovascular diseases	41 (29.5%)
Renal diseases	37 (26.6%)
Cerebrovascular diseases	35 (25.2%)
Solid malignancy	21 (15.1%)
Chronic pulmonary diseases	20 (14.3%)
Hematologic malignancy	16 (11.5%)
Liver diseases	10 (7.2%)
Organ transplantation	4 (2.9%)
HIV infection	2 (1.4%)
Previous hospitalization within 3 months, n (%)	73 (52.5%)
Site of infection in 139 patients at enrollment, n (%)	
Lower respiratory tract infection	93 (66.9%)
Blood stream infection	40 (28.8%)
Urinary tract infection	18 (12.9%)
Gastrointestinal tract infection	11 (7.9%)
Skin and soft tissue infection	8 (5.8%)
Surgical site infection	1 (0.7%)
Bone and joint infection	1 (0.7%)
Documented bacteria-caused infections in 110 patients that required colistin therapy, n (%)	
*A. baumannii*	79 (71.8%)
*K. pneumoniae*	18 (16.4%)
*P. aeruginosa*	9 (8.2%)
*E. coli*	2 (1.8%)
*E. cloacae*	2 (1.8%)
Medical procedure, n (%)	
Mechanical ventilation	117 (84.2%)
Central venous catheterization	85 (61.2%)
Chronic intermittent hemodialysis	53 (38.1%)
Severity of patient illness, mean ± SD	
APACHE II score at enrollment	21.8 ± 7.4
Antibiotic use for the current hospitalization prior to study enrollment, n (%)	134 (96.4%)
Carbapenems	112 (80.6%)
Cephalosporins	56 (40.3%)
Beta-lactam/beta-lactamase inhibitors	75 (54.0%)
Fluoroquinolones	39 (28.1%)
Vancomycin	43 (30.9%)
Aminoglycosides	6 (4.3%)
Colistin	9 (6.8%)
Colistin use within 3 months prior to study enrollment, n (%)	15 (10.8%)

The mean age of subjects was 67.2 years, and the distribution between genders was not different. Most subjects had chronic underlying diseases or healthcare-associated conditions. Previous hospitalization within 3 months was observed in 52.5% of subjects. Lower respiratory tract infection was the most common site of infection, followed by blood stream infection and urinary tract infection. Colistin therapy was given to 79.1% of subjects with documented bacterial infections, and in 77.1% of episodes of documented bacterial infection. *A. baumannii* was the most common cause of documented infection, followed by *K. pneumoniae* and *P. aeruginosa*. *E. coli* and *Enterobacter cloacae* were found in only 4% of subjects. Most subjects received mechanical ventilation (84.2%) or central venous catheterization (61.2%). The mean APACHE II score was 21.8. Most patients received various antibiotics within 3 months prior to study enrollment, including carbapenems, cephalosporins, beta-lactam/beta-lactamase inhibitors, fluoroquinolones, vancomycin, aminoglycosides and colistin. The subjects received 166 courses of colistin during the study period. Colistin was given to 110 subjects (79.1%) with documented infections, with 29 patients (20.9%) being prescribed colistin as empirical therapy. The most frequent indication for colistin use was *A. baumannii* ventilator-associated pneumonia. The median duration of all colistin treatment courses was 9 days (range: 1–55).

### Colistin-resistant *E. coli* or *K. pneumoniae*

The surveillance culture samples collected included 673 stool or rectal swab samples, 465 sputum samples, 103 urine samples, 2 percutaneous drainage samples, and 1 wound swab sample. Colistin resistance was observed in 246 isolates of EC and KP, of which 26 were EC and 220 were KP. One hundred and sixty-six isolates (67.5%) of CoR EC or CoR KP were recovered from stool or rectal swab samples, while 70 isolates (28.5%) and 10 isolates (4.1%) were recovered from sputum samples and urine samples, respectively. The MIC_50_, MIC_90_, and MIC range of all CoR EC and CoR KP isolates was 64, 128, and 4 to > 128 mg/L, respectively. The MIC_50_, MIC_90_, and MIC range of all CoR EC isolates was 8, 64, and 4 to 128 mg/L, respectively. The MIC_50_, MIC_90_, and MIC range of all CoR KP isolates was 64, 128, and 4 to > 128 mg/L, respectively. Among the 26 CoR EC isolates, 80.8% were resistant to ceftriaxone, and 11.5% were resistant to carbapenems. Ceftriaxone resistance and carbapenems resistance was observed in 96.8 and 75.5% of 220 CoR KP isolates, respectively.

### Colistin-resistant *E. coli* or *K. pneumoniae* colonization in all collected clinical samples

**Fig. 1:**
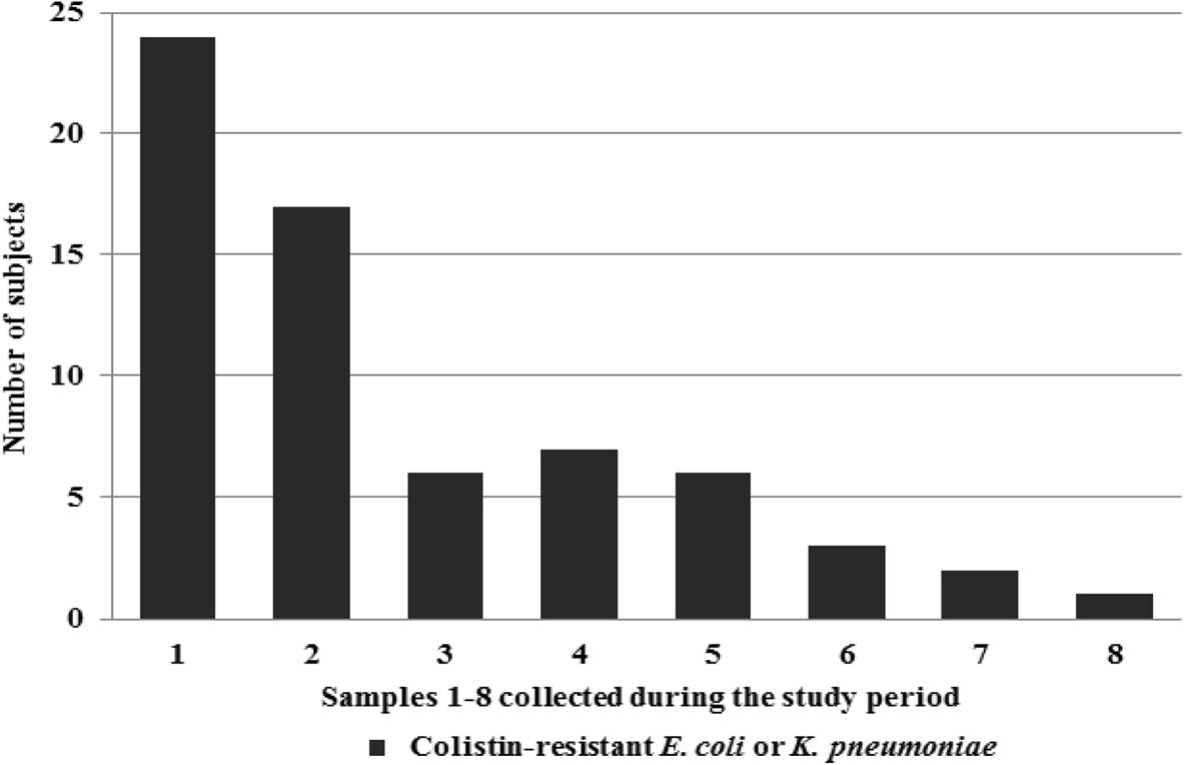
Number of subjects colonized with colistin-resistant *E. coli* or *K. pneumoniae* in surveillance culture samples at each of 8 sample collections during the study period

The number of subjects colonized with CoR EC or CoR KP in the first surveillance culture samples at study enrollment, and the number of patients colonized with CoR EC or CoR KP in subsequent surveillance culture samples during the study period are shown in Fig. 1.

CoR EC or CoR KP colonization was detected in 66 of 139 subjects (47.5%). Sixty of those patients (43.2%) were colonized with CoR KP, and 13 subjects (9.4%) were colonized with CoR EC. CoR EC or CoR KP colonization was detected in 24 subjects (17.3%) at study enrollment, with 22 subjects (15.8%) colonized by CoR KP and 2 subjects (1.4%) colonized by CoR EC. Of the 115 subjects with no colonization of CoR EC or CoR KP at study enrollment, 42 subjects (36.5%) subsequently developed colonization with CoR EC or CoR KP. Of those, 39 subjects (33.9%) were colonized with CoR KP, and 8 (7.0%) were colonized with CoR EC.

### Comparison of subjects with and without colistin-resistant *E. coli* or *K. pneumoniae* colonization during the study period

Comparison of subjects with (66 patients) and without (73 patients) CoR EC or CoR KP colonization during the study period is shown in Table 2.

**Table 2:** Comparison of subjects with and without colistin-resistant *E. coli* or *K. pneumoniae* colonization during the study period

Variables	Colonization (*n* = 66)	No Colonization (*n* = 73)	*p*-value
Female gender (yrs), n (%)	33 (50.0%)	37 (50.7%)	0.94
Age (yrs), mean ± SD	65.7 ± 16.7	68.5 ± 15.1	0.32
Duration from hospital admission to first sample collection (days), median (range)	16 (1–106)	16 (2–81)	0.37
Duration from enrollment to first sample collection (days), median (range)	1 (1–4)	1 (1–5)	0.54
ICU admission, n (%)	24 (36.4%)	24 (32.9%)	0.67
Previous hospitalization, n (%)	36 (54.5%)	37 (50.7%)	0.65
APACHE II score at study enrollment, mean ± SD	21.6 ± 7.1	21.9 ± 7.6	0.77
Mechanical ventilation, n (%)	59 (89.4%)	58 (79.5%)	0.11
Indwelling central venous catheter, n (%)	45 (68.2%)	40 (54.8%)	0.11
Surgery, n (%)	45 (68.2%)	39 (53.4%)	0.08
Chronic intermittent hemodialysis, n (%)	8 (12.1%)	10 (13.7%)	0.78
Antibiotic use for the current hospitalization prior to study enrollment and during study period, n (%)			
Carbapenems	57 (86.4%)	66 (90.4%)	0.46
Cephalosporins	31 (47.0%)	31 (42.5%)	0.59
Beta-lactam/ beta-lactamase inhibitors	54 (81.8%)	52 (71.2%)	0.14
Fluoroquinolones	42 (63.6%)	33 (45.2%)	0.03
Vancomycin	40 (60.6%)	38 (52.1%)	0.31
Aminoglycosides	10 (15.2%)	2 (2.7%)	< 0.001
Antibiotic use for the current hospitalization prior to study enrollment, n (%)			
Carbapenems	52 (78.8%)	60 (82.2%)	0.61
Cephalosporins	26 (39.4%)	30 (41.1%)	0.84
Beta-lactam/beta-lactamase inhibitors	37 (56.1%)	38 (52.1%)	0.64
Fluoroquinolones	24 (36.4%)	15 (20.5%)	0.04
Vancomycin	22 (33.3%)	21 (28.8%)	0.56
Aminoglycosides	5 (7.6%)	1 (1.4%)	0.10
Colistin use within 3 months prior to study enrollment, n (%)	13 (19.7%)	2 (2.7%)	0.001
Total amount of colistin (mg), median (range)	3550 (400–12,550)	2000 (500–10,650)	< 0.001
Total duration of colistin use (days), median (range)	13.5 (2–58)	9 (2–32)	< 0.001
Total courses of colistin therapy, mean ± SD	1.4 ± 0.8	1.1 ± 0.4	0.003
Total courses of colistin therapy, range	1–4	1–3	
Patients who received more than 1 course of colistin therapy, n (%)	19 (28.8%)	7 (9.6%)	0.004
Length of hospital stay (days), median (range)	46.5 (12–246)	32 (6–192)	0.01
All-cause mortality at hospital discharge, n (%)	35 (53.0%)	35 (47.9%)	0.55

The duration from hospital admission to the first surveillance culture sample collection, and the duration from study enrollment to the first surveillance culture sample collection during the study period were not significantly different between groups. The use of fluoroquinolones, aminoglycosides, or colistin within 3 months prior to study enrollment, and the amount, duration, and courses of colistin were all significantly greater in subjects with CoR EC or CoR KP colonization than in those without CoR EC or CoR KP colonization (all *p* < 0.05). The median and range of duration of the presence of CoR EC or CoR KP colonization was 13 days and 1–68 days, respectively. Neither CoR EC nor CoR KP was isolated from 23 subjects (34.8%) at the end of the study period, whereas 43 patients (65.2%) had persistent CoR EC or CoR KP colonization at the end of the study period. The median and range of duration of colistin use in subjects that had no CoR EC or CoR KP colonization at the end of the study was 11 days and 2–57 days, respectively. The corresponding values for patients who had persistent CoR EC or CoR KP colonization at the end of the study were 14 days and 3–65 days, respectively (*p* = 0.43). The length of hospital stay in subjects with CoR EC or CoR KP colonization was significantly longer than in subjects without CoR EC or CoR KP colonization. The rates of all-cause mortality at hospital discharge were similar between subjects with and without CoR EC or CoR KP colonization.

### Comparison of subjects with and without colistin-resistant *E. coli* or *K. pneumoniae* colonization at study enrollment

CoR EC or CoR KP colonization was detected in 24 subjects (17.3%) at study enrollment. Twenty-two subjects (15.8%) were colonized with CoR KP, and 2 patients (1.4%) were colonized with CoR EC. Comparison of subjects with and without CoR EC or CoR KP colonization at study enrollment is shown in Table 3.

**Table 3:** Comparison of subjects with and without colistin-resistant *E. coli* or *K. pneumoniae* colonization at study enrollment

Variables	Colonization at enrollment (*n* = 24)	No colonization at enrollment (*n* = 115)	*p*-value
Female gender	13 (54.2%)	57 (49.6%)	0.68
Age (yrs), mean ± SD	64.3 ± 18.8	67.8 ± 15.2	0.33
ICU admission, n (%)	9 (37.5%)	39 (33.9%)	0.77
Previous hospitalization, n (%)	15 (62.5%)	58 (50.4%)	0.28
Duration from hospital admission to enrollment (days), median (range)	19.5 (4–106)	15 (1–86)	0.10
Duration from enrollment to first sample collection (days), median (range)	1.5 (1–4)	1 (1–5)	0.48
APACHE II score at study enrollment, mean ± SD	20.6 ± 7.2	21.9 ± 7.4	0.65
Mechanical ventilation, n (%)	20 (83.3%)	97 (84.3%)	1.0
Indwelling central venous catheter, n (%)	17 (70.8%)	68 (59.1%)	0.29
Surgery, n (%)	19 (79.2%)	65 (56.5%)	0.04
Chronic intermittent hemodialysis, n (%)	4 (16.7%)	14 (12.2%)	0.74
Colistin use within 3 months prior to study enrollment, n (%)	4 (16.7%)	11 (9.6%)	0.47
Antibiotic use for the current hospitalization prior to study enrollment, n (%)			
Carbapenems	20 (83.3%)	92 (80.0%)	0.79
Cephalosporins	9 (37.5%)	47 (40.9%)	0.76
Beta-lactam/beta-lactamase inhibitors	16 (66.7%)	59 (51.3%)	0.17
Fluoroquinolones	13 (54.2%)	26 (22.6%)	0.002
Vancomycin	13 (54.2%)	30 (26.1%)	0.007
Aminoglycosides	3 (12.5%)	3 (2.6%)	0.06
Colistin	3 (13.6%)	6 (5.5%)	0.36
Length of hospital stay (days), median (range)	49.5 (13–138)	36 (6–246)	0.17
All-cause mortality at hospital discharge, n (%)	16 (66.7%)	54 (47.0%)	0.08

Duration from hospital admission to study enrollment, and duration from study enrollment to first surveillance culture sample collection between subjects with and without CoR EC or CoR KP colonization were not significantly different between groups. The use of fluoroquinolones or vancomycin for the current hospitalization prior to study enrollment and surgery were significantly more prevalent in subjects with CoR EC or CoR KP colonization at study enrollment than in those without CoR EC or CoR KP colonization at study enrollment. The use of aminoglycosides for the current hospitalization prior to study enrollment and colistin use within 3 months prior to study enrollment were observed more often in subjects with CoR EC or CoR KP colonization at study enrollment. The median and range of the duration of the presence of CoR EC or CoR KP colonization were 18.5 days and 2–68 days, respectively. CoR EC or CoR KP was not isolated from 25.0% of 24 subjects at the end of the study period. In contrast, the remaining 75.0% of those subjects had persistent CoR EC or CoR KP colonization at the end of the study period. Length of hospital stay and the rate of all-cause mortality at hospital discharge in subjects with CoR EC or CoR KP colonization at study enrollment were both greater than in patients without CoR EC or CoR KP colonization at study enrollment.

### Comparison of subjects with and without colistin-resistant *E. coli* or *K. pneumoniae* colonization after study enrollment among subjects without colonization by colistin-resistant *E. coli* or *K. pneumoniae* at study enrollment

Of the 115 subjects without colonization of CoR EC or CoR KP at study enrollment, 42 patients (36.5%) subsequently developed colonization with CoR EC or CoR KP. Comparison of subjects with and without CoR EC or CoR KP colonization after study enrollment among subjects without colonization by CoR EC or CoR KP at study enrollment is shown in Table 4.

**Table 4:** Comparison of subjects with and without colistin-resistant *E. coli* or *K. pneumoniae* colonization after study enrollment among subjects without colonization with colistin-resistant *E. coli* or *K. pneumoniae* at study enrollment

Variables	Colonization after enrollment (*n* = 42)	No Colonization after enrollment (*n* = 73)	*p*-value
Female gender (yrs), n (%)	20 (47.6%)	37 (50.7%)	0.75
Age (yrs), mean ± SD	66.6 ± 15.6	68.5 ± 15.1	0.54
ICU admission, n (%)	15 (35.7%)	24 (32.9%)	0.76
Previous hospitalization, n (%)	21 (50.0%)	37 (50.7%)	0.94
APACHE II score at study enrollment, mean ± SD	21.8 ± 7.2	21.9 ± 7.6	0.92
Mechanical ventilation, n (%)	39 (92.9%)	58 (79.5%)	0.06
Indwelling central venous catheter, n (%)	28 (66.7%)	40 (54.8%)	0.21
Surgery, n (%)	26 (61.9%)	39 (53.4%)	0.38
Chronic intermittent hemodialysis, n (%)	4 (9.5%)	10 (13.7%)	0.51
Antibiotic use for current hospitalization prior to study enrollment and during study period, n (%)			
Carbapenems	35 (83.3%)	66 (90.4%)	0.26
Cephalosporins	20 (47.6%)	31 (42.5%)	0.59
Beta-lactam/beta-lactamase inhibitors	33 (78.6%)	52 (71.2%)	0.39
Fluoroquinolones	25 (59.5%)	33 (45.2%)	0.14
Vancomycin	23 (54.8%)	38 (52.1%)	0.78
Aminoglycosides	5 (11.9%)	2 (2.7%)	0.10
Antibiotic use for the current hospitalization after study enrollment, n (%)			
Carbapenems	19 (45.2%)	34 (46.6%)	0.89
Cephalosporins	6 (14.3%)	4 (5.5%)	0.17
Beta-lactam/beta-lactamase inhibitors	25 (59.5%)	36 (49.3%)	0.29
Fluoroquinolones	19 (45.2%)	25 (34.2%)	0.24
Vancomycin	17 (40.5%)	27 (37.0%)	0.71
Aminoglycosides	4 (9.5%)	2 (2.7%)	0.19
Colistin use within 3 months prior to study enrollment, n (%)	9 (21.4%)	2 (2.7%)	0.002
Total amount of colistin (mg), median (range)	2800 (400–9350)	2000 (300–10,650)	0.03
Total duration of colistin use (days), median (range)	13 (2–55)	9 (1–32)	0.03
Total courses of colistin therapy, mean ± SD	1.3 ± 0.6	1.1 ± 0.34	0.02
Total courses of colistin therapy, range	1–3	1–3	
Patients who received more than 1 course of colistin therapy, n (%)	10 (23.8%)	6 (8.2%)	0.02
Length of hospital stay (days), median (range)	46.5 (12–246)	32 (6–192)	0.04
All-cause mortality at hospital discharge, n (%)	35 (47.9%)	19 (45.2%)	0.78

Duration from hospital admission to the first surveillance culture sample collection, and duration from study enrollment to the first surveillance culture sample collection in patients with and without CoR EC or CoR KP colonization after study enrollment were not significantly different between groups. The use of colistin within 3 months prior to study enrollment, and the amount, duration, and courses of colistin were all significantly greater in patients with CoR EC or CoR KP colonization after study enrollment than in those without CoR EC or CoR KP colonization after study enrollment. The median and range of the time from study enrollment to the first detection of CoR EC or CoR KP colonization were 10 days and 3–42 days, respectively, and the mean duration was 14 days for CoR KP and 14 days for CoR EC. The median and range of the duration of the presence of CoR EC or CoR KP colonization was 7 days and 1–57 days, respectively. At the end of the study, CoR EC or CoR KP was and was not isolated from 59.5 and 40.5% of 42 subjects, respectively. Length of hospital stay in subjects with CoR EC or CoR KP colonization after study enrollment was significantly longer than in subjects without CoR EC or CoR KP colonization after study enrollment. The rate of all-cause mortality at hospital discharge was similar between subjects with and without CoR EC or CoR KP colonization after study enrollment.

### Presence of mcr-1 gene among colistin-resistant *E. coli* and *K. pneumoniae* isolates

The mcr-1 gene was detected in 13.0% of 246 isolates of CoR EC or CoR KP. It was detected in 57.7% of 26 CoR EC isolates, while only 7.7% of 220 CoR KP isolates showed presence of mcr-1. The colistin MIC of CoR EC isolates with the mcr-1 gene ranged from 4 to 16 mg/L (median: 8 mg/L), whereas the colistin MIC of CoR KP isolates with the mcr-1 gene ranged from 4 to > 128 mg/L (median: 32 mg/L). All isolates of CoR EC or CoR KP with the mcr-1 gene were colonizers. Presence of the mcr-1 gene was observed in 27.3% of the 66 subjects that had CoR EC or CoR KP. The mcr-1 gene was detected in 12.5% of 24 subjects with CoR EC or CoR KP at study enrollment, and in 35.7% of 42 subjects with colonization with CoR EC or CoR KP after study enrollment.

### Colistin-resistant *E. coli* or *K. pneumoniae* infection

Five subjects had infections caused by CoR KP. Of those, three had infections at study enrollment, and 2 developed infections after the start of the study. Of the 3 subjects with infections caused by CoR KP at study enrollment, 2 had concurrent CoR KP colonization. Of the 2 subjects with infections caused by CoR KP after study enrollment, 1 had concurrent CoR KP colonization and the other had concurrent CoR EC colonization. The sites of infections were urinary tract infections (3 patients), ventilator-associated pneumonia (1 patient) and bacteremia (1 patient). All subjects received colistin in combination with either fosfomycin or aminoglycoside, and they were alive at hospital discharge. There was no infection caused by CoR EC among subjects who had CoR EC colonization throughout the study period.

## Discussion

The overall CoR EC or CoR KP colonization rate of 47.5% among subjects who received colistin in the present study was relatively high, especially the development of CoR EC or CoR KP colonization after study enrollment when compared with data from previous reports [15–17]. However, the study populations in those previous reports were general hospitalized patients, and they did not limit their studies to only patients who received colistin. To the best of our knowledge, the present study is the first to examine the prevalence of CoR EC or CoR KP colonization among hospitalized patients who received colistin. The prevalence of CoR EC or CoR KP in surveillance culture samples collected from these subjects was also high. It should be noted that a much larger number of CoR KP isolates than CoR EC isolates was recovered from subjects in this study (220 isolates vs. 26 isolates, respectively).

Many factors may have contributed to the development of CoR EC and CoR KP colonization observed in this study. The subjects with CoR EC or CoR KP colonization at study enrollment may have acquired colistin-resistant bacteria from the community prior to hospitalization. The probability of community-acquired CoR EC or CoR KP is low, however, because it is extremely rare for individuals, foods, and the environment in the community in Thailand to harbor CoR EC or CoR KP [18, 19]. CoR EC and CoR KP have been detected at food animal farms in Thailand (especially swine farms) that use colistin extensively in the feed given to the swine to prevent and treat infection (V. Thamlikitkul. unpublished data). Most of the colistin-resistant Enterobacteriaceae found at swine farms in Thailand was *E. coli* while the prevalence of CoR EC isolates observed in this study was much lower than that of CoR KP isolates. A study from China reported the presence of colistin-resistant Enterobacteriaceae in healthy adults [20]. Subjects with CoR EC or CoR KP colonization at study enrollment in the present study may have had colonized CoR EC or CoR KP in their guts from prior hospitalizations. The transmission of CoR EC or CoR KP from other hospitalized patients with colonization or infections caused by these bacteria and/or from the hospital environment to subjects during hospitalization prior to study enrollment could not be excluded. The use of fluoroquinolones, vancomycin, or aminoglycosides, all of which are non-beta-lactam antibiotics, for the current hospitalization prior to study enrollment may have contributed to the development of CoR EC or CoR KP colonization at study enrollment, because these antibiotics were found to be or tended to be associated with subjects who had CoR EC or CoR KP colonization at study enrollment. Interestingly, the use of beta-lactams during the current hospitalization prior to study enrollment was not found to be significantly associated with CoR EC or CoR KP colonization at study enrollment. Colistin use within 3 months prior to study enrollment may also have contributed to the development of CoR EC or CoR KP colonization at study enrollment, although potential statistical significance could not be achieved due to the small size of our study population. CoR EC or CoR KP colonization could have developed in some subjects in the present study after exposure to colistin for only 3 days, which would be consistent with our previous observations in swine that showed colistin-resistant Enterobacteriaceae colonization in the guts of piglets that received colistin for only several days. Association between the emergence of CoR EC or CoR KP and the use of broad-spectrum antibiotics and colistin was previously reported [3, 11, 17, 21]. Colistin-resistant bacteria in patients with no history of colistin use was also reported [22–24].

In patients with no CoR EC or CoR KP colonization at study enrollment that developed CoR EC or CoR KP colonization after study enrollment, it appears evident that colistin use was the main contributing factor, because colistin use within 3 months prior to study enrollment and a larger total dose of colistin, longer duration of colistin, and multiple courses of colistin were all significantly associated with subjects who developed CoR EC or CoR KP colonization after study enrollment. In addition to the use of colistin, transmission of CoR EC or CoR KP from other hospitalized patients with colonization or infection with these colistin-resistant bacteria and/or from the hospital environment during hospitalization could also be potential causes of CoR EC or CoR KP colonization. Since the present study did not have a concurrent cohort of hospitalized patients who did not receive colistin, the effect and magnitude of these other potential causes of CoR EC or CoR KP colonization (in addition to colistin exposure) could not be quantified. Our previous study in CRE colonization and infections in hospitalized patients at our center revealed that approximately 10% of patients had colistin-resistant Enterobacteriaceae during their hospitalization (V. Thamlikitkul. unpublished data). Therefore, colistin use should still be considered a major contributing driver of CoR EC or CoR KP colonization in subjects who receive colistin. Two previous studies reported emergence of colistin resistance in *K. pneumoniae* after the use of colistin for digestive tract decontamination [25, 26]. Selective pressure of colistin use in these patients is likely to be one of the contributing factors for this emergence of colistin resistance.

CoR EC or CoR KP was not isolated from 34.8% of subjects with CoR EC or KP colonization at the end of the study period. The median and range of the duration of CoR EC or CoR KP colonization in these subjects were 18.5 days and 2–68 days, respectively. It should be kept in mind that collection of surveillance culture samples was not performed on a daily basis, but they were collected at enrollment, on days 3 and 7 after the start of colistin treatment, and once a week thereafter. As such, the reported duration from the first sample collection until the disappearance of CoR EC or CoR KP does not represent the real duration of colonization. The duration of colonization could be shorter than the reported duration since CoR EC or CoR KP could disappear between the sample collection times. These observations indicate that colistin resistance could be disappeared, which is similar to colistin resistance findings in Enterobacteriaceae studies in piglets that rapidly developed resistance when colistin was given, but the resistance was usually disappeared once colistin was discontinued (V. Thamlikitkul. unpublished data). Regarding subjects with persistent CoR EC or CoR KP colonization at the end of the study period, colonization duration data was not available since these patients were not followed beyond the end of the study period. The duration of colistin exposure in patients with persistent CoR EC or CoR KP colonization at the end of the study seemed to be longer than in those with no observable CoR EC or CoR KP colonization at the end of the study.

The mechanisms of colistin resistance in Enterobacteriaceae should be different from those in non-fermentative Gram-negative bacteria, especially *A. baumannii* and *P. aeruginosa*. *A. baumannii* and *P. aeruginosa* are far less likely to develop colistin resistance during exposure to colistin, because the prevalence of colistin-resistant *A. baumannii*-caused and *P. aeruginosa*-caused infections in hospitalized patients at Siriraj Hospital has been less than 5% even though colistin has been widely used for treatment of carbapenem-resistant *A. baumannii* and *P. aeruginosa* infections at Siriraj Hospital for longer than 10 years [27]. In contrast, brief exposure by *E. coli* and *K. pneumoniae* to colistin in hospitalized patients was associated with colistin resistance in *E. coli* and *K. pneumoniae*, as was observed in the present study and in the previous study that investigated colistin use in swine.

Although the number of CoR KP isolates was far larger than the number of CoR EC isolates in our study population, the prevalence of the mcr-1 gene in CoR KP isolates (7.7%) was much lower than in CoR EC isolates (57.7%). This observation was similar to observations from our previous study that the mcr-1 gene was detected in only 1.4% of 280 suspected CoR KP isolates, whereas it was detected in 29.7% of 37 suspected CoR EC isolates [28]. Again, similar to data from our previous study, the MICs of colistin in CoR EC isolates with the mcr-1 gene were lower than the colistin MICs in CoR KP isolates with the mcr-1 gene [28]. Since the mcr-1 gene was detected in only some isolates of CoR EC and in only a small portion of CoR KP isolates, CoR EC and CoR KP without the mcr-1 gene isolated from subjects in our study could be resistant to colistin via other types of plasmid-mediated mcr genes (including mcr-2, mcr-3, mcr-4, mcr-5), or other resistance mechanisms, or combined mechanisms of resistance [29–33]. We are currently investigating the mechanisms of colistin resistance among CoR EC and KP isolates without the mcr-1 gene.

CoR EC or CoR KP colonization in hospitalized patients could pose multiple threats to their own wellbeing and the wellbeing of others. Although this study found only 3 subjects who had CoR KP colonization and infection, hospitalized patients with CoR EC or CoR KP colonization should be considered to be at risk of developing infections due to CoR EC or CoR KP at a later time point. This later risk of infection was observed in neutropenic patients, in other immunocompromized patients or critically-ill patients who developed subsequent multidrug-resistant infections after colonization with the same multidrug-resistant bacteria, and surveillance culture collected from such patients to determine the presence of multidrug-resistant colonization was suggested because intestinal colonization with multidrug-resistant bacteria was found to be associated with subsequent infections caused by the same colonized multidrug-resistant bacteria [34–37]. Hospitalized patients with CoR EC or CoR KP colonization could transmit these colistin-resistant bacteria to other hospitalized patients, healthcare personnel, their relatives, and into the hospital environment. Patients with an antibiotic-resistant plasmid-mediated gene, such as mcr-1, in colonized bacteria could transfer this genetic mobile element to other bacteria residing in their body, other patients, or the hospital environment, which could result in further emergence and spread of colistin-resistant pathogens. Patients with persistent colonization of CoR EC or CoR KP could transmit these bacteria to others living in their home and into the environment in their community when they return home from the hospital, and these colistin-resistant bacteria may cause community-acquired infection in the future. The length of hospital stay in subjects with CoR EC or CoR KP colonization was significantly longer than the length of stay among subjects without CoR EC or CoR KP colonization; however, the all-cause mortality rate at hospital discharge was similar between subjects with and without CoR EC or CoR KP colonization.

The outcomes of infections caused by CoR EC or CoR KP are not always unfavorable or fatal. Many isolates of CoR EC and some isolates of CoR KP recovered from subjects in this study were still susceptible to carbapenems. Although all 5 subjects infected with CoR KP had infections caused by carbapenem-resistant isolates, all of them received combination of colistin and fosfomycin or aminoglycosides and they were alive at hospital discharge. Infection caused by CoR EC was not observed in this study. This may be mostly and simply due to the fact that there was only a small number of subjects who had colonization with CoR EC compared to a much more number of subjects with CoR KP colonization.

Polymyxins will continue to be a mainstay antimicrobial group for therapy of carbapenem-resistant *A. baumannii* and *P. aeruginosa* infections, both of which are very prevalent in hospital-acquired infections in many countries, including Thailand. Polymyxins will also be the treatment of choice for CRE infection, which has been an emerging hospital-acquired infection in Thailand over the past 5 years, since more effective, more efficient, and safer agents for treatment of these carbapenem-resistant Gram-negative infections are not yet available. The responsible physician should prescribe colistin for therapy of documented carbapenem-resistant Gram-negative infections that are susceptible to colistin at the shortest duration of treatment. Colistin should not be used in patients with carbapenem-resistant Gram-negative colonization, and it should be used as empirical antimicrobial therapy as rarely as possible. These strategies for the use of colistin may minimize collateral damage caused by the use of colistin regarding the development and transmission of CoR EC or CoR KP in both healthcare and community settings. However, even appropriate use of colistin is still a key driver of the inevitable emergence of CoR EC or CoR KP. The high prevalence of CoR EC or CoR KP colonization observed in this study is worrisome, and it may predict an increase in CoR EC or CoR KP infections in the future. The heavy use of colistin in hospitalized patients may result in infections caused by CoR EC or CoR KP becoming endemic in healthcare facilities in the near future.

## Conclusions

Prevalence of CoR EC or CoR KP colonization in hospitalized patients receiving colistin was high and it was associated with the use of colistin. Therefore, patients who receive colistin are at risk of developing CoR EC or CoR KP colonization and infection.
